# Do expectancies of return to work and Job satisfaction predict actual return to work in workers with long lasting LBP?

**DOI:** 10.1186/s12891-016-1314-2

**Published:** 2016-11-17

**Authors:** Jon Opsahl, Hege R. Eriksen, Torill H. Tveito

**Affiliations:** 1 Uni Research Health, Postboks 7810, Bergen, 5020 Norway; 2 Buskerud and Vestfold University College, Horten, Norway; 3 Department of Sport and Physical Activity, Bergen University College, Bergen, Norway

**Keywords:** Long lasting low back pain, Chronic low back pain, Expectancies of returning to work, Return to work expectations, Job satisfaction, Return to work

## Abstract

**Background:**

Musculoskeletal disorders including low back pain have major individual and socioeconomic consequences as it often leads to disability and long-term sick leave and exclusion from working life. Predictors of disability and return to work often differ, and the dominant knowledge is on predictors for prolonged sick leave and disability. Therefore it is also important to identify key predictors for return to work. The aim of the study was to assess if overall job satisfaction and expectancies of return to work predicts actual return to work after 12 months, among employees with long lasting low back pain, and to assess if there were gender differences in the predictors.

**Methods:**

Data from the Cognitive interventions and nutritional supplements trial (CINS Trial) was used. Predictors for return to work were examined in 574 employees that had been on sick leave 2–10 months for low back pain, before entering the trial. Data were analysed with multiple logistic regression models stratified by gender, and adjusted for potential confounders.

**Results:**

Regardless of gender high expectancies were a strong and significant predictor of return to work at 12 months, while high levels of job satisfaction were not a significant predictor. There were no differences in the levels of expectancies or overall job satisfaction between men and women. However, men had in general higher odds of returning to work compared with women.

**Conclusions:**

Among individuals with long lasting low back pain high expectancies of returning to work were strongly associated with successful return to work. We do not know what factors influence individual expectancies of return to work. Screening expectancies and giving individuals with low expectancies interventions with a goal to change expectancies of return to work, such as CBT or self-management interventions, may contribute to increase actual return to work.

**Trial registration:**

http://www.clinicaltrials.gov/, with registration number NCT00463970. The trial was registered at the 18th of April 2007.

## Background

Musculoskeletal complaints are the single largest category of work related illness and it accounts for a third or more of all registered occupational diseases in the Nordic countries, USA, and Japan [1]. Musculoskeletal complaints is the predominant cause of sick leave and disability benefits in Norway, and low back pain (LBP) is the largest single diagnosis in this group [2, 3]. LBP causes more global disability than any other condition [3], and in Norway the direct and indirect costs related to LBP are estimated to 13–15 billion NOK yearly [4].

Returning to work after sick leave due to long lasting LBP is often a valued goal for a working adult. Scientific evidence indicate that work is both a fundamental determinant and a prerequisite for health [5], and that work has beneficial effects not only on mental and physical health, but also on well-being [6]. Most people with low back pain are working, and for the majority there are no clear cut or absolute reasons for not working even when symptomatic [6].

Key predictors of return to work (RTW) need to be identified to find effective RTW interventions that may infer positive individual and societal effects. The literature on prediction of occupational outcomes such as RTW has to a large extent been within the pathogenic paradigm, focusing on those at risk for disability rather than those who do RTW [7]. The reality that predictors of disability and predictors of RTW often differ [7] further underlines the importance of finding the key predictors of RTW.

Heitz et al. (2009) identified a host of significant prognostic factors predicting RTW, 44 biomedical (27 modifiable) and 61 psychosocial (40 modifiable) in chronic LBP [8]. Several researchers have underlined the need to decrease the growing list of workplace variables to a feasible set of core factors [9]. Summarized evidence from five systematic reviews identified seven core factors, where each of the core factors where at least supported by one of the reviews [9]. The seven core factors were: (1) heavy physical demands, (2) ability to modify work, (3) job stress, (4) social support, (5) job satisfaction, (6) RTW expectation, (7) fear of re-injury [9]. Of the work characteristics reviewed so far, it has been argued that job satisfaction undoubtedly has the highest statistical correlate with health [10].

Differences in prognostic factors on LBP for RTW between men and women have previously been found [11]. However, only a few studies have presented gender specific analyses [11, 12]. To our knowledge this is the first study reporting results from gender specific analyses on prognostic factors in a cohort of individuals with long lasting low back pain. In turn, this might have implications for who and which factors to address.

The Cognitive Activation Theory of Stress is a theory which points out the importance of expectancies [13]. Whenever an individual is faced with a new task, challenge, demand or threat, activation occurs. The outcome of this activation depends on the individual response outcome expectancies. Response outcome expectancies depend on previous learning, and within CATS, coping is defined as positive response outcome expectancies, and leads to a temporary activation that may have a positive influence on health [13]. Establishing positive response outcome expectancies may also increase the individual efforts to solve the task or reduce the threat [14]. In this case, the threat may be the LBP. Workers with sub-acute or chronic LBP with a previous back pain episode have shown higher RTW rates and shorter periods of disability than workers without a previous episode [15]. It may be argued that they have experienced that when they have an episode of low back pain, it is likely to be temporary and thus the outcome will be positive. Similar findings have been shown in organizational change, where employee's previous learning experiences are associated with positive attitudes towards organizational change [16].

Recovery expectations has been identified as one of two most consistent predictors for RTW across several statistical models [17]. Positive recovery expectancies have also been associated with decreased pain and improved functional status [17]. However, less than 30% of the LBP population in the study by Schultz et al. [17] had chronic LBP, and it has been demonstrated that the number of modifiable prognostic factors are higher in acute- and sub-acute samples with LBP than chronic LBP [8]. These arguments suggest that it may be important to investigate the role of recovery expectancies in a large sample of patients with long lasting LBP.

The aim of the current study was to assess if overall job satisfaction and expectancies of return to work predicts actual return to work after 12 months, among employees with long lasting low back pain, and to assess if there were gender differences in the predictors.

## Methods

The analyses in this study were conducted with data from the Cognitive Interventions and Nutritional Supplements trial (CINS trial) (ref Reme et al. 2011 for details of the CINS trial [18]). Permission to use the data was obtained from the principal investigator of the CINS trial, Hege Randi Eriksen. The CINS trial was a randomized controlled trial (RCT), which examined the effectiveness of a brief intervention (BI) and compared it to BI and cognitive behavioral therapy (CBT), BI plus soy oil, and BI plus seal oil. In the CINS trial 160 participants were recruited to two different sub-studies of the main trial. These participants are also included in this study.

### Participants

In this study 634 participants with long lasting LBP were assessed for eligibility and 574 were included in the study, 49.7% were men, and the mean age was 44.3 years (SD 9.7), (see Table 1).

**Table 1:** Characteristics of the study population (*N* = 569)

	Men (*n* = 283)	Women (*n* = 286)	*t* (df) or *χ*2 (df)	*p*-value*
Sociodemographic factors				
Age, *M (SD)*	44.3 (9.7)	44.3 (9.7)	.055 (567)	0.956
Gender	49.7%	50.3%		
Education:			20.493 (4)	**<0.001**
Primary and secondary	17.6%	10.4%		
Upper secondary	52.6%	44.6%		
College/University 1–4 years	17.3%	26.6%		
College/University ≥ 4 years	4.4%	11.2%		
Other	8.1%	7.2%		
Smoking (yes)	46.5%	38.2%	4.254 (1)	**0.039**
Covariates				
FABQ-Work, *M (SD)*	25.7 (9.6)	24.1 (10.3)	1.914 (547)	0.56
SHC, *M (SD)*	15.7 (9.5)	19.3 (9.4)	−4.360 (521)	<0.001
ODI, *M (SD)*	28.8 (12.3)	29.3 (12.6)	−408 (550)	0.684
HSCL:			4.556 (1)	**.033**
HSCL - < 1.75	70.6%	62%		
HSCL - ≥ 1.75	29.4%	38%		
Co-worker social support, *M (SD)*	19.3 (3.3)	18.8 (3.3)	1.971 (547)	**0.049**
Predictor variables				
Job satisfaction:			5.162 (3)	0.160
Very satisfied	32.3%	29.2%		
Satisfied	45%	45.8%		
Neither satisfied or dissatisfied	18.2%	15.90%		
Dissatisfied	3%	7.6%		
Very dissatisfied	1.5%	1.4%		
Return to work expectancies:			6.814 (3)	0.078
High expectancies	73.3%	75.1%		
Moderate expectancies	13.3%	17.3%		
Low expectancies	6.6%	2.9%		
Do not know	7%	4.7%		
Outcome				
Work status at 12 months:				
Returned to work	60.1%	52.4%		

Continuous variables are presented by means (*M*) with standard deviation (*SD*) in parentheses, and categorical variables by percentages. *N* refers to the total sample size, and may deviate in some of the variables due to missing data

* Statistical tests and *p*-value for gender differences

Note: *χ* ^2^- tests between gender and the outcome, “Work status at 12 months”, were not performed as the associations between the independent variables and the dependent variable were analyzed with logistic regression models

The inclusion criteria were: 1) sick leave due to LBP for 2–10 months; 2) at least 50% sick listed; 3) both participant and physician agreed that randomization was acceptable; 4) written informed consent from the participants; 5) at least 50% employed; 6) one of the following ICPC diagnoses: L02, L03, L84, or L86; 7) age between 20 and 60 years. The exclusion criteria were: 1) less than 50% sick listed or not on sick leave anymore; 2) pregnancy; 3) hemophilia; 4) osteoporosis (known osteoporotic fracture, or on anti-osteoporotic medication); 5) currently being treated for cancer; 6) recent back trauma; 7) serious psychiatric disorders (mainly due to ongoing psychosis, high suicide risk, and/or serious depression), assumed to be incompatible with participation in the trial; 8) not fluent in Norwegian (assumed to be incompatible with CBT); 9) debilitating cardiovascular disease; 10) on warfarin treatment (blood thinner, e. g. Marevan); 11) ongoing insurance trial, lawsuit, or pending legal action for LBP or related conditions.

**Fig. 1:**
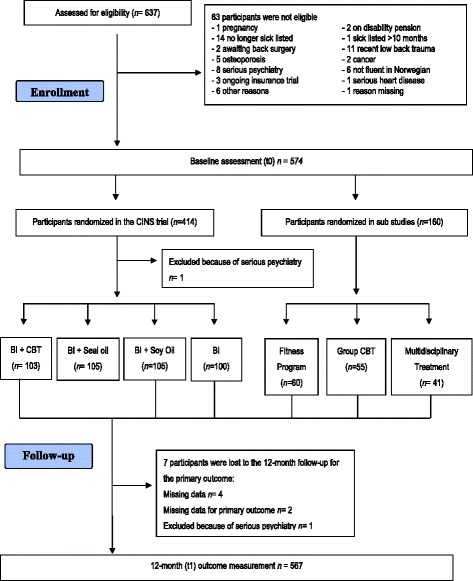
Flow chart

The flow chart of the study is shown in Fig. 1.

### Measures

Potential prognostic factors were assessed by means of the CINS questionnaire at baseline assessment (t0) which included Norwegian versions of instruments, covering a broad range of factors including demographic variables, physical variables, individual- and work related psychological variables and social support.

### Instruments

#### Job satisfaction

Job satisfaction was measured with the single-question from Quinn and Shepard [19], “all in all, how satisfied or dissatisfied with your job?” translated into Norwegian. The item was rated on a five point Likert scale, ranging from 1 - very dissatisfied to 5 - very satisfied [19]. According to an meta analysis reviewing the quality of single-item questions measuring job satisfaction, single-questions have shown convergent validity with multi-item scales [20].

#### Expectancies of returning to work

The study participants were asked about their own expectancies of RTW with one single question; “*To what extent do you think you will return to work*?” (translated by the authors). The item was rated on a four point scale, 1 - ‶*to a low degree*‶ (low expectancies); 2 - ‶*to a certain degree*” (moderate expectancies)”; 3 – “*to a high degree*” (high expectancies); 4 – “*do not know*”.

#### Covariates

A multitude of factors have been found to increase the risk of developing LBP and the risk for a prolonged course of LBP. In an attempt to reduce the risk of alternative explanations several variables with previously demonstrated associations between long lasting LBP and return to work were adjusted for in the analyses. The covariates included sociodemographic factors; age, education and smoking. Other covariates which were included were; fear avoidance beliefs (FAB) about LBP, subjective health complaints, disability, emotional distress, and co-worker social support. We also included the intervention groups as a covariate [18, 21].

#### Fear avoidance beliefs

Fear-avoidance beliefs were measured with the *Fear-avoidance beliefs questionnaire (FABQ)* [22, 23]. The FABQ consists of two subscales; the five item fear avoidance beliefs for physical activity (FABQ-PA) and the 11 item fear avoidance beliefs for work (FABQ-Work). Each item is scored on a 7-point Likert scale ranging from 0 – “completely disagree” to 6 – “completely agree”. Higher scores on the questionnaire as a whole, or either of the subscales, indicate increased fear-avoidance beliefs [23]. The Norwegian version of the FABQ has displayed reliability almost equal to the English version for the two subscales [24]. Only the FABQ-Work subscale was used as it has been shown to be a better predictor of self-reported disability and work loss in patients with chronic LBP compared to the FABQ-PA [25, 26].

#### Subjective health complaints (SHC)

SHC were measured with The SHC Inventory [27] containing 29 items of ordinary somatic and psychological complaints. The participants were asked to rate the intensity of each complaint experienced during the last 30 days on a four-point scale; 0 – “not at all”, 1 – “a little”, 2 – “some”, 3 – “severe”. A total score of SHC was computed by summing the score on all the 29 items. The total score was used to indicate the degree of co-morbid health complaints, and high scores on subjective health complaints are associated with high levels of sick leave [28]. The questionnaire has been tested and has demonstrated acceptable reliability and validity [27].

#### Disability

The Oswestry Disability Index (ODI) version 2.0 was used to assess disability [29, 30]. The ODI contains 10 items which assess activity limitations; pain intensity, personal hygiene, lifting, walking, sitting, standing, sleeping, sexual activity, social activity, and travelling. Each item was scored on a six-point scale; 0 – “no limitation” to 5 – “maximal limitation” [30]. The total score was calculated as suggested by Fairbank and Pynsent [30] (total score/(5 x number of questions answered))/100%, giving a score range of 0–100. ODI has shown acceptable reliability and construct validity for assessing functional status of Norwegian-speaking patients with LBP [31].

#### Co-worker social support

The social support subscale of the Demand-Control-Support-Questionnaire (DCSQ) was used to measure co-worker social support [32]. The co-worker social support subscale consists of six items. Each item is scored on a four-point scale ranging from 1 – “completely true” to 4 –“completely untrue”. High score indicates increased co-worker social support. The Norwegian version of the co-worker social support subscale has demonstrated satisfactory reliability [33].

#### Emotional distress

Symptoms of emotional distress were measured with the Hopkins Symptom Check List-25 (HSCL-25) [34, 35]. The HSCL-25 consists of 25 items and each item is rated on a four-point scale; 1 – “not at all” to 4 – “extremely”. The total score is the mean of all 25 items The cut-off score equal to or greater than 1.75 was used to define “a case” [35].

### Analyses

All statistical analyses were performed with SPSS version 19 for Windows. Differences between men and women at baseline (t0) were assessed with two-way t-tests and *χ* ^2^ tests. The categorical variables which violated the assumption of the *χ* ^2^- tests were recoded; smoking was recoded into a dichotomous smoker/non-smoker variable. The smoker category included all smokers, from smoking on a daily basis to smoking less than once a week as even the all-cause mortality is higher in intermittent male smokers compared with non-smoking men [36].

For RTW expectancies “low expectancies” and “moderate expectancies” needed to be merged in order to not violate the assumption of the goodness-of-fit tests in logistic regression (observed and expected cell count no more than 20% less than 5). The categories “very dissatisfied” and “dissatisfied” were merged for overall job satisfaction.

Bivariate and multiple logistic regression analyses were stratified by gender and used to assess the association between the predictor variables and the outcome, and the covariates and the outcome. To assess the impact of gender on return to work, non-stratified bivariate and multiple logistic regression analyses were also performed. The results from the logistic regression analyses are presented as odds ratios (OR) with 95% confidence interval (CI).

If the crude estimates for the predictor variables were significant for the outcome, multivariate logistic regression analyses were performed. Further, in the multivariate logistic regression analyses all the covariates with *p*-values < .10 for either men or women were adjusted for in the model. However, age was kept regardless of the *p*-value due to common practice [15, 37], and that lower age is predictive of RTW in long lasting LBP [38]. In the multivariate logistic regression analyses expectancies of returning to work and the covariates were entered in the following six blocks: I) return to work expectancies; II) age and sociodemographic factors; III) Intervention group, IV) FABQ-Work, SHC, and ODI; V) emotional distress; VI) co-worker social support. No multivariate analyses were performed for global job satisfaction for either men or women because *p* >0.10, (men *p =* 0.433, women *p =* 0.16).

For explained variance Nagelkerke R square was used.

## Results

### Characteristics of the study population

319 (56.3%) of the patients had returned to work at 12 months follow up, with slightly more men than women (see Table 1). The level of education was low, and there were a high number of smokers in the sample. Men had significantly lower education, were more likely to smoke, reported lower level of SHC, and emotional distress compared to women (see Table 1).

### Predictors of RTW at 12 month follow up

High levels of job satisfaction did not predict RTW at 12 months for either men or women.

Having high expectancies of returning to work predicted RTW at 12 months for both men and women, while having uncertain expectancies were no better than having low or moderate expectancies (see Table 2).

**Table 2:** Prospective effects of the predictor variables and the covariates on RTW at 12 months. Crude estimates from bivariate logistic regression analyses

Variables	Categories ^a^	Men		Women	
		*OR*	*95% CI*	*OR*	*95% CI*
Expectancies of returning to work	[Low or moderate expectancies]	ref			
	High expectancies	5.38	2.81–10.33	4.80	2.47–9.35
	Do not know	1.27	0.42–3.79	1.33	0.35–5.01
Job satisfaction	[Very dissatisfied or dissatisfied]	ref			
	Neither satisfied or dissatisfied	0.74	0.21–2.67	0.68	0.25–1.84
	Satisfied	1.09	0.33–3.62	1.07	0.45–2.52
	Very satisfied	1.36	0.40–4.64	2.17	0.87–5.39
Gender	^a^	1.51	1.19–1.92	^a^	^a^
Age		1.01	1.00–1.01	1.00	0.996–1.01
Highest completed education	[Primary and secondary]	ref			
	Upper secondary	1.45	1.04–2.02	1.10	0.77–1.57
	College/University 1–4 years	1.19	0.67–2.13	1.74	1.08–2.79
	College/University >4 years	1.40	0.44–4.41	1.21	0.60–2.46
	Other	3.40	1.25–9.22	0.25	0.08–0.75
Smoking status	[Smokers]	ref			
	Non-smokers	1.64	1.17–2.29	1.06	0.79–1.43
Co-worker social support	[Low Support]	ref			
	Moderate support	1.58	0.95–2.64	1.29	0.86–1.92
	High Support	2.16	1.41–3.31	1.07	0.71–1.61
FABQ-Work	[High FAB]	ref			
	Moderate FAB	1.41	0.92–2.14	1.54	1.02–2.33
	Low FAB	3.63	2.19–6.03	1.90	1.23–2.94
SHC	[High SHC]	ref			
	Moderate SHC	1.12	0.74–1.70	1.02	0.68–1.53
	Low SHC	2.46	1.53–3.95	1.87	1.22–2.88
ODI	[High disability]	ref			
	Moderate disability	2.24	1.45–3.47	0.98	0.65–1.46
	Low disability	2.07	1.32–3.25	1.82	1.20–2.77
HSCL-25	[HSCL - ≥ 1.75]	ref			
	HSCL - < 1.75	1.7	1.27–2.28	1.19	.88–1.61

Note: Reference categories in brackets

^a^ Reference category for gender = women

In the unadjusted model expectancies of returning to work explained 15.4 and 12.4% of the variance seen in RTW for men and women respectively. In the fully adjusted model the explained variance seen in RTW increased to 33.4 and 28.3% for men and women respectively. Further, the initial associations between return to work expectancies were mildly attenuated by adjustments of the covariates (see Table 3). Expectancies of returning to work classified almost 90% of those who did return to work correctly.

**Table 3:** Prospective effects of high expectancies of returning to work on RTW at 12 months. Multivariable logistic regression analyses with cumulative adjustments for potential confounding factors

Adjustment Variables	Men			Women		
	*OR*	*95% CI*	*Nagelkerke R* ^*2*^	*OR*	*95% CI*	*Nagelkerke R* ^*2*^
No adjustment	5.38	2.81–10.33	.154	4.80	2.47–9.35	.124
+ age, sociodemographic factors^a^	4.53	2.21–9.28	.183	4.31	2.13–8.73	.160
+ intervention groups	4.52	2.19–9.32	.191	4.46	2.18–9.12	.181
+ FABQ-Work, SHC total, ODI	4.04	1.86–8.76	.333	3.29	1.55–6.97	.280
+ Emotional distress (HSCL-25)	4.11	1.88–8.97	.334	3.36	1.58–7.14	.283
+ Co-worker social support	4.17	1.90–9.17	.334	^b^	^b^	^b^

^a^ Highest completed education, smoking status

^b^ Not included

Note: Reference category = low or moderate expectancies of RTW

### Gender differences

Men had higher odds of returning to work compared to women in the non-stratified bivariate analyses. In the fully adjusted non-stratified model, men had 1.57 higher odds (95% CI 1.03–2.40) of RTW compared to women.

A greater number of the covariates contributed significantly to the prediction of RTW for men compared to the women, including high perceived co-worker social support, being a non-smoker, and not reporting emotional distress (see Table 2).

## Discussion

Among both men and women with long lasting low back pain, having high expectancies of returning to work was a significant and strong predictor for returning to work at 12 months follow-up. After adjusting for sociodemographic factors and other covariates, men and women with high expectancies for return to work had 3 to 5 times higher odds of returning to work compared to their respective counterparts with low or moderate expectancies of RTW. The small effect of the covariates on the strength of the relation between high expectancies and RTW is in line with findings from a systematic review on expectancies and health outcomes [39]. The results from the present study are also in line with several studies on acute and sub-acute LBP, all demonstrated that high expectancies predicted RTW [17, 40–42].

Expectancies have an intuitive influence on RTW, however it is argued that there is too little evidence to firmly conclude that expectancies have a significant effect on RTW [43]. On the other hand, only two out of the five previously mentioned studies which are consistent with the present results were included in the systematic review by Fadyl and McPherson [43].

The Cognitive Activation Theory of Stress (CATS) [13] provides an explanation for the reason why the subjects reporting high expectancies of returning to work have a strong and significant increased probability of returning to work compared to those with low or moderate expectancies. Having high expectancies of returning to work can be considered as coping within CATS. This implies that the individual has acquired positive response outcome expectancies. This leads the individual to use whatever strategy he or she places the highest confidence in for solving the problem [27], i.e. RTW. Interestingly it has been found that workers with sub-acute/chronic LBP with a previous back pain episode had higher RTW rates than workers without a previous episode. It did also predict shorter disability [15, 37]. This is in line with the postulated position of coping in the CATS, the individual learns from his or her previous experience, and the experience of returning to work reinforces the individual positive response outcome expectancies with a high perceived probability for future RTW.

The explained variation of the crude RTW model was slightly higher for men (15.4%) compared to women (12.4%). After adjusting for possible confounders the explained variance of the RTW model increased to more than 30% for both genders. This indicated that RTW is influenced by the prognostic factors in this study. Still, more than 2/3 of the variance in RTW is still left unexplained. However, expectancies of returning to work classified almost 90% of those who did return to work correctly. This implies that expectancies play an important role for successful RTW. Since the majority of the study participants reported high expectancies of returning to work, interventions solely aimed to target expectancies of returning to work may have limited beneficial effects on increased RTW rates. Adding other predictors that encompass key aspects of the complex environment of occupational health care may also be important.

A cross cultural comparison between western countries of RTW after chronic LBP, found that the eligibility criteria for entitlement to long term and/or partial disability benefits contributed to the differences in sustainable RTW [44]. The authors found that less strict compensation policies to be eligible for long term (partial) benefits were more effective in achieving sustainable RTW. From a health promotion perspective, it would therefore be interesting if the explained variation in RTW may be raised by adding system or contextual obstacles to the model such as whether the public policy promotes RTW or if it is actually a barrier.

Surprisingly, being very satisfied or satisfied with the job did not predict RTW at 12 months for either the men or the women. In previous studies assessing relationships between job satisfaction and return to work it seems that the pathogenic paradigm has been dominant, predicting the risk of non-return to work or disability [45, 46]. The results from these studies are ambiguous, and a study of Norwegian industry workers found a positive association between low level of job satisfaction and long-term sick leave [47]. This finding is backed up by one systematic review which highlighted that job dissatisfaction predicted non-return to work or disability with strong level of evidence [45]. However, another systematic review drew the complete opposite conclusion that there is strong evidence that job satisfaction is not predictive of work outcome in non-chronic LBP [46].

The results from present study is in discordance with a study by van der Giezen, Bouter and Nijhuis [38] who found that higher level of job satisfaction independently predicted RTW.

Although the results from this study for overall job satisfaction was non-significant with regards to return to work, this does not necessarily mean that job satisfaction is unimportant. From a broader health perspective a large meta-analysis clearly demonstrated that the level of job satisfaction is an important factor influencing the health of workers, e.g. dissatisfied workers are more likely to experience emotional burn-out, and to have raised symptom levels of anxiety and depression [10].

In this sample, when comparing the responses from men and women regarding expectancies of returning to work and global job satisfaction, almost identical percentages were found for the different response categories. There were no statistical significant associations between gender and these two constructs. This indicates that men and women reported almost the same level of expectancies of returning to work and global job satisfaction. These results are in accordance with a study by A Sousa-Poza and AA Sousa-Poza [48] who did not find any gender differences in job satisfaction. On the other hand, in the fully adjusted model men with high expectancies of returning to work had a higher OR of returning to work compared to women. This in some ways surprising since women had significantly higher education than men, and more men were smokers compared to women. Previous studies have shown that those with higher education are more likely to RTW after sickness absence due to musculoskeletal complaints, including LBP [49] and that smoking has a strong negative effect on sick leave in a representative working population [50]. Moreover, the result that men were more likely to RTW is in some ways discordant to a study by De Rijk et al. [51] who did not find gender differences in first RTW. On the other hand, in the same study, women reported longer time to lasting RTW compared to men. In addition, RTW is often measured differently across studies [52], and this may also explain different results.

Another explanation is that the women in this sample reported a higher score on the subjective health complaints and a higher proportion reported emotional distress compared to men. It has been demonstrated that comorbidity in workers with LBP increases the likelihood of remaining disabled from work [51]. The result that women reported a significantly higher SHC score is in agreement with findings from the Norwegian general population, where women reported more subjective health complaints compared to men [53]. Suggested explanations of these gender differences includes differences in responses to stress, differences in coping styles, higher total workload, higher pressure with regards to family and career, or plainly that females have a lower threshold for experiencing and reporting complaints [53].

The strengths of this study includes that the data were collected prospectively, obtaining data for the exposure and outcome from different sources, the access to a range of health related information, the large sample size with regards to the cohort under investigation compared with previous studies, the low attrition rate and the gender specific results.

The limitations of the study concern the validity of the predictor variables and the primary outcome measurement and residual confounding. Expectancies of returning to work were measured with an unvalidated scale, which may jeopardize the generalizability of the results if not replicated. However, the majority of previous studies measuring recovery expectancies have also used single-item questions with a Likert scale response rating relating to statement(s) regarding expectancies [43, 54]. This illustrates that the concept lacks a standard and or consistent measure. As a result the best way to measure work related recovery expectancies remains unclear [54].

## Conclusions

Among individuals with long lasting low back pain high expectancies of returning to work were strongly associated with successful return to work. In terms of the practical implications of this study, screening expectancies about returning to work and giving extra attention to those with low expectancies may be useful. Interventions focusing on knowledge and coping, might empower the individual and provide them with “tools” to manage long lasting low back pain, without or with limited need of using health care services. These “tools” may consist of cognitive and behavioral strategies that can be used when needed. For instance cognitive behavioral therapy are recommended in the treatment of long lasting LBP [55, 56]. However, interventions solely focusing on the individual might have limited beneficial effect on achieving better RTW rates since multiple stakeholders are involved in the return to work process. It may be argued that large opportunities in achieving enhanced return to work rates lies within the workplace [52, 57]. This combined with the findings from the present study implies that the most promising return to work interventions might be interventions addressing three key elements; individual psychological factors such as expectancies, work environmental factors [58] and factors related to the involvement of the various stakeholders [59].

## Abbreviations

BI: Brief intervention
CATS: Cognitive Activation Theory of Stress
CBT: Cognitive behavioral therapy
CINS trial: Cognitive Interventions and Nutritional Supplements trial
FABQ: Fear avoidance beliefs questionnaire
LBP: Low back pain
RCT: Randomized controlled trial
RTW: Return to work
